# Diversity Promotes Temporal Stability across Levels of Ecosystem Organization in Experimental Grasslands

**DOI:** 10.1371/journal.pone.0013382

**Published:** 2010-10-13

**Authors:** Raphaël Proulx, Christian Wirth, Winfried Voigt, Alexandra Weigelt, Christiane Roscher, Sabine Attinger, Jussi Baade, Romain L. Barnard, Nina Buchmann, François Buscot, Nico Eisenhauer, Markus Fischer, Gerd Gleixner, Stefan Halle, Anke Hildebrandt, Esther Kowalski, Annely Kuu, Markus Lange, Alex Milcu, Pascal A. Niklaus, Yvonne Oelmann, Stephan Rosenkranz, Alexander Sabais, Christoph Scherber, Michael Scherer-Lorenzen, Stefan Scheu, Ernst-Detlef Schulze, Jens Schumacher, Guido Schwichtenberg, Jean-François Soussana, Vicky M. Temperton, Wolfgang W. Weisser, Wolfgang Wilcke, Bernhard Schmid

**Affiliations:** 1 Max Planck Institute for Biogeochemistry, Hans-Knöll Strasse, Jena, Germany; 2 Department of Special Botany and Functional Biodiversity Research, University of Leipzig, Leipzig, Germany; 3 Hydrology, Institute of Ecology, University of Jena, Jena, Germany; 4 UFZ Helmholtz Center for Environmental Research, Leipzig, Germany; 5 Institute of Geography, Friedrich Schiller University Jena, Jena, Germany; 6 Institute for Plant, Animal and Agroecosystem Sciences, ETH Zurich, Zürich, Switzerland; 7 Soil Ecology, UFZ Helmholtz Centre for Environmental Research, Halle, Germany; 8 Institute of Zoology, Darmstadt University of Technology, Darmstadt, Germany; 9 J.F. Blumenbach Institute of Zoology and Anthropology, Georg-August-University Göttingen, Göttingen, Germany; 10 Institute of Plant Sciences, University of Berne, Berne, Switzerland; 11 Institute of Biochemistry and Biology, University of Potsdam, Potsdam, Germany; 12 Tartu College, Tallinn University of Technology, Tartu, Estonia; 13 Center for Population Biology, Imperial College London, Silwood Park, United Kingdom; 14 Institute of Evolutionary Biology and Environmental Studies, University of Zürich, Zürich, Switzerland; 15 Geographic Institute, Johannes Gutenberg University, Mainz, Germany; 16 Department of Crop Sciences, University of Göttingen, Göttingen, Germany; 17 Faculty for Biology, University of Freiburg, Freiburg, Germany; 18 Grassland Ecosystem Research, Institut National de la Recherche Agronomique, Clermont-Ferrand, France; 19 Phytosphere Institute ICG-3, Forschungszentrum Jülich GmbH, Jülich, Germany; 20 Geographic Institute, University of Berne, Berne, Switzerland; 21 Institute for Stochastics, University of Jena, Jena, Germany; University of Chicago, United States of America

## Abstract

The diversity–stability hypothesis states that current losses of biodiversity can impair the ability of an ecosystem to dampen the effect of environmental perturbations on its functioning. Using data from a long-term and comprehensive biodiversity experiment, we quantified the temporal stability of 42 variables characterizing twelve ecological functions in managed grassland plots varying in plant species richness. We demonstrate that diversity increases stability i) across trophic levels (producer, consumer), ii) at both the system (community, ecosystem) and the component levels (population, functional group, phylogenetic clade), and iii) primarily for aboveground rather than belowground processes. Temporal synchronization across studied variables was mostly unaffected with increasing species richness. This study provides the strongest empirical support so far that diversity promotes stability across different ecological functions and levels of ecosystem organization in grasslands.

## Introduction

Ecosystems are subjected to natural environmental perturbations ranging from small- to large-scale processes. Species-rich communities host a variety of life strategies that can respond differently to environmental perturbations and contribute to ecological functioning in various ways, thus increasing ecosystem stability [1]–[4]. However, the mechanisms by which species-specific variations in response to perturbations translate into ecosystem stability are still debated [5], [6] and it remains largely unknown whether the diversity–stability hypothesis holds for ecological functions other than plant community biomass. In this context, temporal stability is defined as the capacity of an ecosystem to dampen environmental perturbations while retaining the ecological function of interest [1]–[4].

In a recent meta-analysis, Jiang and Pu [7] showed that biodiversity stabilizes community variables and may additionally stabilize species populations in multi-trophic systems. Their result led to the proposition that in the presence of temporal synchronization —when different variables respond similarly to environmental perturbations— stabilization of ecological functions at the component level (e.g., population) of organization may promote stabilization at the system level (e.g., community). This proposition contrasts with previous theories that predicted populations to be destabilized and communities to be stabilized [4], [8], but is in agreement with other conceptual models [9]–[11]. Acknowledging a possible bottom-up effect of species richness on the temporal stability of ecological functions at the community level of organization, two corollaries follow. First, species richness decreases the temporal variance of ecological functions at both the component level of *species* (populations of single species or groups of functionally similar species) and the system level of the *community* (plant community or ecosystem property). Second, the temporal co-variance between variables characterizing an ecological function (e.g., between the biomass production of plant functional groups, such as herbs, grasses and legumes, contributing to the aboveground plant biomass production) is positive and not affected by species richness. In other words, ecological functions at lower (population) levels of organization may be stabilized by species richness and, if variables co-vary positively in time, this stabilizing effect would propagate through the whole ecosystem. However, a critical evaluation of the above two corollaries can only be accomplished with an experimental approach. Furthermore, in spite of recent meta-analyses indicating that ecological functioning is enhanced by higher biodiversity [12]–[14], it is not clear whether biodiversity concurrently increases the temporal stability of these functions across many levels of organization. We analyzed this question for multiple ecological functions measured in a single experiment.

Using 7 years of data collected on 82 large plots in a grassland biodiversity experiment (the Jena Experiment) [15], [16], we tested to what degree higher species richness of plant communities translates into temporal stability when functions are characterized at the organizational level of the population, the functional and phylogenetic group, the community, or the ecosystem (Table 1). Naturally-occurring or management-caused environmental perturbations in the Jena Experiment (50°57′3.09″N, 11°37′23.49″E) include summer droughts, winter soil freezing, mowing of the vegetation twice a year, and spatial heterogeneity in vegetation cover through weeding. In this experiment, the lower resistance of less diverse communities against spontaneously invading species was shown to occur independently of the spatial heterogeneity in vegetation cover [17]. Functions at different organizational levels refer to: the species level (populations of species), the functional and phylogenetic level (groups of similar species), the community level of primary producers and secondary consumers, and the ecosystem level (mainly biogeochemical soil properties).

**Table 1:** Summary of 42 variables used to characterize the stability of twelve ecological functions.

Ecological function	Variable	Units	Time extent	Obs: Plots	Field protocol
Earthworm Biomass	*Lumbricus terrestris*	g/m^2^	2005–2008 ^i^	6∶45	Octett method
	*Aporrectodea caliginosa*	g/m^2^	2005–2008 ^i^	6∶45	Octett method
**POPULATION**	*Aporrectodea rosea*	g/m^2^	2005–2008 ^i^	6∶45	Octett method
Parasitic	Pteromalidae sp.	Count	2003; 2005 ^s^	10∶50	Suction sample
Hymenoptera	Ceraphronidae sp.	Count	2003; 2005 ^s^	10∶50	Suction sample
	Diapriidae sp.	Count	2003; 2005^ s^	10∶50	Suction sample
**POPULATION**	Encyrtidae sp.	Count	2003; 2005^ s^	10∶50	Suction sample
Invasive Plant	[1/*Chenopodium album*]	g/m^2^	2002–2004^ i^	6∶67	2.5×2.0 m quadrat
Bioregulation	[1/*Sonchus asper*]	g/m^2^	2002–2004 ^i^	6∶67	2.5×2.0 m quadrat
**POPULATION**	[1/*Taraxacum officinale*]	g/m^2^	2002–2004 ^i^	6∶67	2.5×2.0 m quadrat
	[1/*Mercurialis annua*]	g/m^2^	2002–2004 ^i^	6∶67	2.5×2.0 m quadrat
Below-Ground	Chilopoda (large predators)	Count	2004–2008 ^i^	7∶82	Kempson soil core
Invertebrates	Coleoptera (small predators)	Count	2004–2008 ^i^	7∶82	Kempson soil core
**PHYLOGENETIC**	Oligochaeta (large prey)	Count	2004–2008 ^i^	7∶82	Kempson soil core
Above-Ground	Diptera (mainly saprophagous)	Count	2003; 2005 ^s^	10∶50	Suction sample
Invertebrates	Heteroptera (predators)	Count	2003; 2005 ^s^	10∶50	Suction sample
**PHYLOGENETIC**	Hymenoptera (mainly parasitic)	Count	2003; 2005 ^s^	10∶50	Suction sample
Plant Functional	Grasses (Poales)	g/m^2^	2003–2008 ^i^	12∶22	0.2×0.5 m quadrat
Group Biomass	Herbs (mainly Asterids)	g/m^2^	2003–2008 ^i^	12∶22	0.2×0.5 m quadrat
**PHYLOGENETIC**	Legumes (Fabales)	g/m^2^	2003–2008 ^i^	12∶22	0.2×0.5 m quadrat
Plant Stand	Biomass (Sown species)	g/m^2^	2003–2008 ^i^	12∶82	0.2×0.5 m quadrat
Structure	Cover (Sown species)	%	2003–2008 ^i^	12∶82	3×3 m quadrat
**COMMUNITY**	Leaf Area Index	m^2^/m^2^	2003–2008 ^i^	12∶82	LAI-2000
	Mean Plant Height	cm	2005–2008 ^i^	8∶82	10 m transect
Invasive Plant	[1/Biomass (Weed species)]	g/m^2^	2002–2007 ^i^	11∶82	0.2×0.5 m quadrat
Bioregulation	[1/Cover (Weed species)]	%	2002–2007 ^i^	11∶82	3×3 m quadrat
**COMMUNITY**	[1/Weeded fresh biomass]	g/m^2^	2002–2007 ^i^	11∶82	2.5×2.0 m quadrat
	[1/Weeded Species Richness]	Count	2002–2004 ^i^	6∶82	2.5×2.0 m quadrat
Arthropod Diversity	Ground Abundance	Count	2003; 2005 ^s^	10∶50	Pitfall trap
	Ground Spp. Richness	Count	2003; 2005 ^s^	10∶50	Pitfall trap
**COMMUNITY**	Aboveground Abundance	Count	2003; 2005 ^s^	10∶50	Suction sample
	Aboveground Spp. Richness	Count	2003; 2005 ^s^	10∶50	Suction sample
Soil Water Content	[1/Soil Moisture 10 cm]	m^3^/m^3^	2008 ^s^	18∶80	FDR
	[1/Soil Moisture 20 cm]	m^3^/m^3^	2008 ^s^	18∶80	FDR
**ECOSYSTEM**	[1/Soil Moisture 30 cm]	m^3^/m^3^	2008 ^s^	18∶80	FDR
	[1/Soil Moisture 40 cm]	m^3^/m^3^	2008 ^s^	18∶80	FDR
Soil Nutrient	[1/Soil Nitrate 15 cm]	µg	2002–2007 ^i^	11∶82	Soil extractions
Concentration	[1/Soil Ammonium 15 cm]	µg	2003–2007 ^i^	9∶82	Soil extractions
**ECOSYSTEM**	[1/Soil Nitrate 30 cm]	µg	2002–2004 ^i^	6∶82	Soil extractions
Trace Gas Fluxes	CO_2_ fluxes	µmol d^−1^ m^−2^	2007–2008 ^s^	6∶78	PVC dark chambers
	N_2_O fluxes	µmol d^−1^ m^−2^	2007–2008 ^s^	6∶78	PVC dark chambers
**ECOSYSTEM**	CH_4_ fluxes	µmol d^−1^ m^−2^	2007–2008 ^s^	6∶78	PVC dark chambers

‘Obs: Plots’ gives the number of measurements recorded across the reported time extent (temporal observations) and the number of assembled species mixtures in which the variables were measured (experimental plots). Superscript letters next to the time extent indicate whether the temporal dynamics of a variable was predominantly seasonal (s) or inter-annual (i). Each ecological function is represented in a multivariate space by three or four field variables, each variable characterizing a different facet of that function at one level of organization (in capital bold letters). Prior to the analyses, each variable was linearly scaled to remove the effect of measurement units.

Kolasa and Li [9] demonstrated that habitat specialization usually increases with species richness and, because the abundance of habitat-specialist species is generally more variable over time, this may mask the stabilizing effect of other species. Here we equated specialization with low abundance and thus excluded sub-dominant species, as well as sub-dominant functional or phylogenetic groups of species; a procedure compatible with Kolasa and Li's formalism. Our approach has the main advantage that the same set of target variables is used to evaluate the effect that varying plant species richness has on temporal stability for different ecological functions and levels of organization (see Materials and Methods).

As a measure of temporal stability [3], [4], we calculated the multivariate coefficient of variation (CV^2^) by summing the variances and co-variances of scaled variables characterizing the same ecological function over time as follows (see Materials and Methods): CV^2^ =  [Σ Variances +Σ Co-variances] / [Σ Means]^2^. The reciprocal of the numerator reflects the first part of the temporal stability definition of the introduction paragraph: “the capacity of an ecosystem to dampen environmental perturbations…”. The denominator reflects the second part of that definition: … “ while retaining the ecological function of interest.” A comparatively lower sum of variances scaled by the square of the summed means (hereafter the variance CV) among variables characterizing an ecological function indicates higher stability, since it implies a dampening of response to a common set of environmental perturbations. Whereas a comparatively lower sum of co-variances scaled by the square of the summed means (hereafter the co-variance CV) among these variables also indicates higher stability since it implies that different facets of an ecological function synchronize less over time, whereby minimizing the risk of functional breakdown. From the above approach, the population is characterized by variables A, B, C measured at the species level, while the community is characterized by another set of variables D, E, F measured at the community level. This notably contrasts with previous approaches [3], [4], [9] where the community level variable D is the sum of A, B, and C.

Our methodology aims at determining how plant species richness influences the temporal variances and co-variances of target variables grouped according to their level of organization and ecological function. However, the CV^2^ measure of variability rests on the mathematical postulate that the variance and the squared mean of a measured variable scale positively and linearly [18]. When more than one variable is used to characterize an ecological function, both the summed variance and co-variance components of the CV^2^ measure are expected to scale linearly with the squared sum of means. More specifically, if the variables are either positively or negatively correlated with one another, the summed co-variances (expressed in absolute terms) reflect the summed variances and both components scale with the squared sum of means. On the other hand, if variables are de-correlated (independent) of one another, the summed co-variances tend towards zero and only the summed variances scale with the squared sum of means. Thus, to be a valid comparative measure of temporal stability, the CV ratio must account for the fact that summed variances and co-variances are not mathematically independent of the summed means. In our study, the use of variance and co-variance CV ratios was justified on two points. i) For all twelve ecological functions, at the alpha rejection rate of 0.05, we found a significantly positive Pearson's r correlation across experimental plots between the squared sum of means and both the summed variances and the summed co-variances expressed in absolute terms. The mean ± 1SD correlation across the twelve ecological functions was 0.743±0.136 (min 0.484, max 0.923) for the variances and 0.525±0.235 (min 0.230, max 0.849) for the co-variances. ii) The slope of the log-log relationships between summed variances (or summed co-variances) and the squared sum of means approached one, indicating that a vast majority of correlations were linear. The mean ± 1SD linear slope across the twelve ecological functions was 0.767±0.183 (min 0.398, max 1.081) for the variances and 0.873±0.236 (min 0.4284, max 1.128) for the co-variances.

## Results

Our synthesis of the diversity–stability hypothesis in the Jena Experiment emphasizes two main results. Firstly, ecological functions at various organizational levels were stabilized with increasing plant species richness, as indicated by a decrease of the variance CV for the abundance of parasitic hymenoptera (food-web complexity), the suppression of non-resident plant species (bioregulation), vegetation structure and biomass production (primary producers), the abundance and diversity of invertebrates (secondary consumers), as well as trace gas fluxes (ecosystem properties). We found that the variance CV of ecological functions processes (Production of Earthworm Biomass and Below-Ground Invertebrates, Soil Nutrient and Water Content) associated to belowground processes was not significantly decreased with increasing species richness.

**Figure 1:**
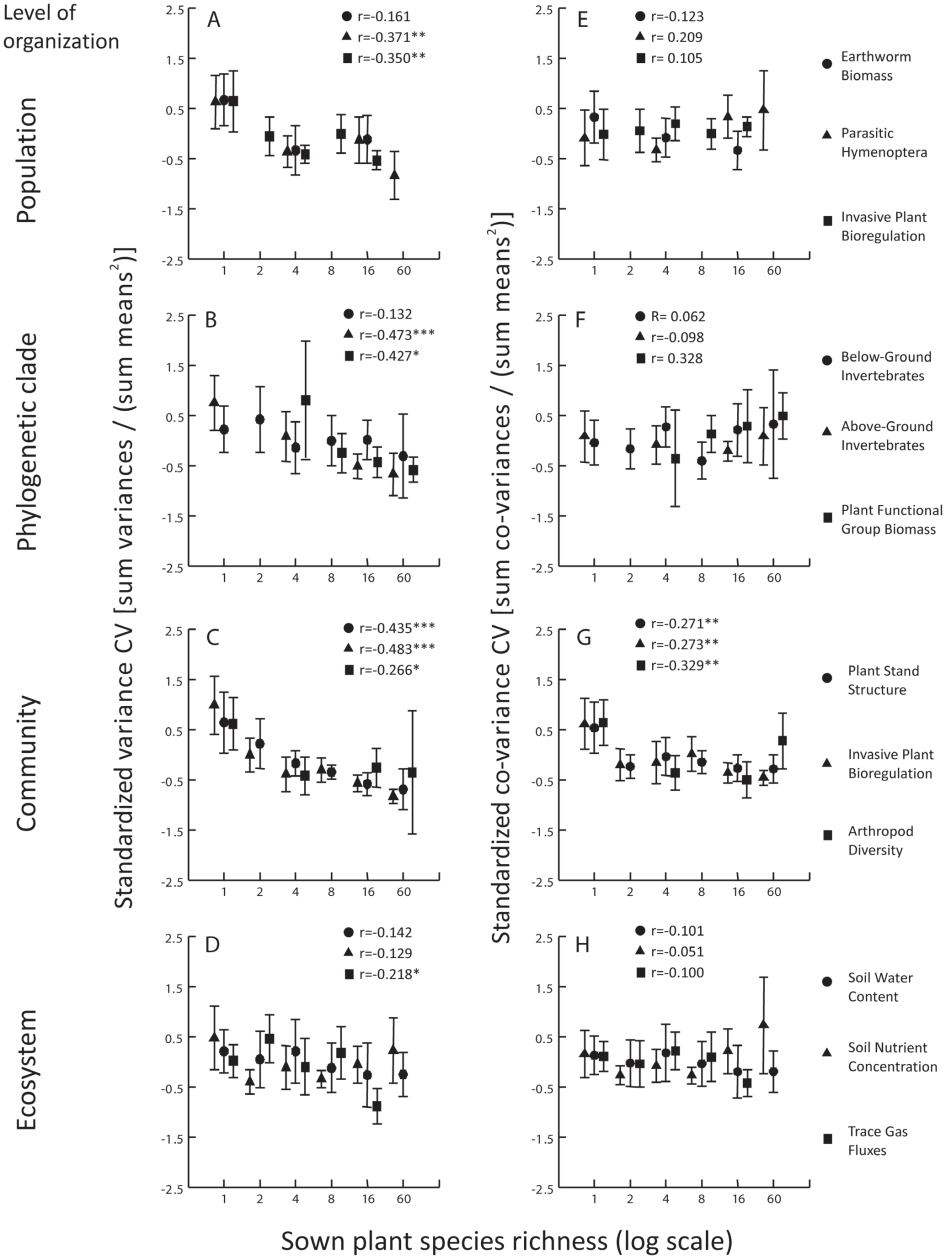
Diversity–stability relationships for the twelve ecological functions grouped by levels of organization. Each dot represents the mean of temporal stability values (vertical axes) obtained across experimental plots sowed with the same number of plant species (1, 2, 4, 8, 16 or 60 species). Error bars represent the 95% confidence interval around the mean. Each temporal stability measure was standardized with a mean of zero and variance of one to ease the comparison of diversity–stability relationships within and between organizational levels. The left panels (A–D) show the relationships between plant species richness and the variance CV, while the right panels (E–H) show the relationships to the co-variance CV. No measurements were available for species richness treatments 2 and 8 in Earthworm Biomass, Parasitic Hymenoptera, Below- and Aboveground Invertebrates, and Arthropod Diversity production functions. For each component of temporal stability we report Pearson's correlation coefficient r estimated by a linear fit between the logarithm of plant species richness and one component of temporal stability. The number of stars next to Pearson's r values gives the probability of accepting the null hypothesis following a distribution-free randomization test [37] (df are in Table 1): Blank (p>0.05), *(p<0.05), **(p<0.01), ***(p<0.001).

**Figure 2:**
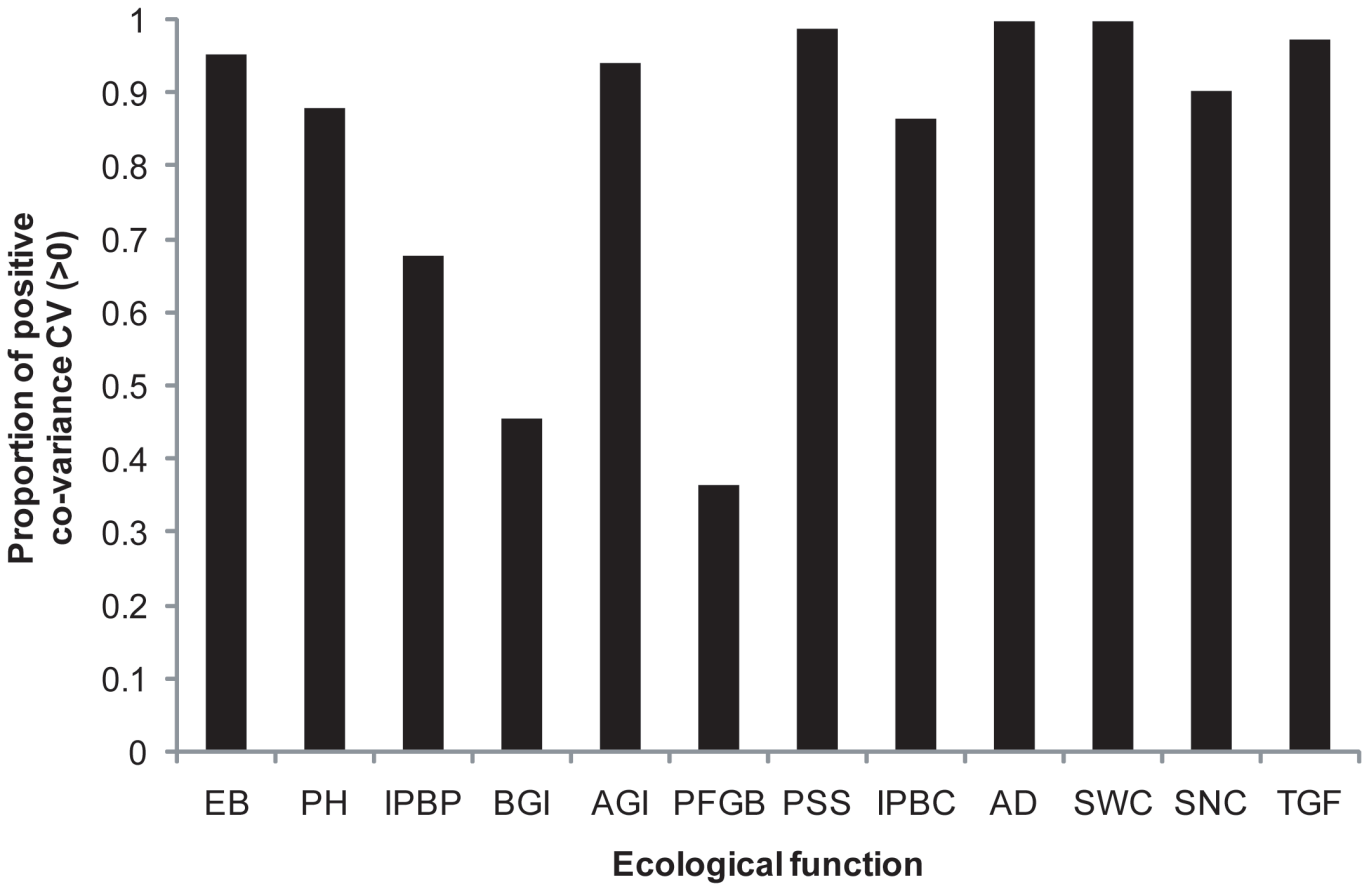
Proportion of positive co-variance CV across experimental plots in each ecological function. EB: Earthworm Biomass; PH: Parasitic Hymenoptera; IPBP: Invasive Plant Bioregulation of Populations; BGI: Below-Ground Invertebrates; AGI: Above-Ground Invertebrates; PFGB: Plant Functional Group Biomass; PSS: Plant Stand Structure; IPBC: Invasive Plant Bioregulation of Communities; AD: Arthropod Diversity; SWC: Soil Water Content; SNC: Soil Nutrient Concentration; TGF: Trace Gas Fluxes. See Table 1 for additional details on the number of experimental plots in each ecological function.

Secondly, the co-variance CV of a majority (9 out of 12) of ecological functions did not show a relationship with species richness. However, the variables characterizing ecological functions at the community level were more synchronized (more dependent on one another) at low than high species richness, as indicated by the increased co-variance CV in monocultures (Figure 1). Furthermore, the proportion of positive co-variance CV across all ecological functions and experimental plots attained 85% (Figure 2), suggesting that temporal synchronization among variables is common.

## Discussion

Our finding that the variance CV of ecological functions decreased with increasing species richness corroborates previous finding about the stabilization of aboveground productivity by biodiversity [3], [19]–[21] and extends it to further functions. We furthermore show that the diversity–stability relationship holds across multiple ecological functions in a single experiment, which is remarkable considering the spectrum of organizational (from population to ecosystem) and trophic levels (from primary producers to higher consumers) investigated. Our results lend support to the proposition that temporal stabilization at the lower levels of organization can promote stabilization at the higher levels (communities, ecosystems) [7], [21]. Some examples from our experiment illustrate this point in more detail. The temporal stability of parasitic wasps could depend on the continuous presence of a diversity of hosts, which in turn would depend on the vegetation structure and the biomass of plant functional groups. Temporal stability against plant invasion by non-resident species may be linked to resource pre-emption; i.e., the environmental resources are exploited by the resident plant species. At the sub-community level, the temporal stability of aboveground invertebrate abundances could be associated with specific plant–insect interactions. Temporal stability in arthropod biodiversity may represent a circumstance where the diversity in grassland plant species promotes diversity at other trophic levels [22].

Unfortunately, our data did not allow disentangling among these alternative hypotheses because time series between ecological functions were either too short or not temporally aligned. Nevertheless, the general picture suggests that species groups that are dominant in the plant community can exert a bottom-up control on the stability of aboveground ecological functions at higher organizational levels. A recent meta-analysis [12] reported that out of sixteen candidate hypotheses, the organizational level had the strongest influence in explaining the magnitude of the biodiversity effect on ecological functions. Our finding that multiple ecological functions are concurrently stabilized by plant species richness is a strong indication that identifying diversity–stability mechanisms requires a complete overview of the identities of at least all dominant species and ecological interactions within and between organizational levels.

Previous studies on grassland systems suggested that diversity promotes temporal stability at the community level, but that the stability of individual species within communities may show the opposite trend [3], [19], [20], [23]. Our study is particular in the sense that, at organizational levels below the community, we considered only variables measured on the dominant populations of species and the dominant groups of functionally similar species. Our results suggest that the increasing response of the variance CV for plant species biomass reported in earlier studies may have been due to the accumulation of habitat specialists at low abundance with increasing species richness [9]. In plant communities, the accumulation of specialists is paralleled by a reduction in the individual species biomass with increasing richness, which follows from the partitioning of total biomass among species per unit area. The variance CV of a few dominant plant species may in fact decrease with increasing species richness, but this stabilizing effect would be confounded by the opposite response of a larger number of sub-dominant species. This interpretation is also in agreement with the “weak interaction effect”, which states that the dynamics of dominant, strongly interacting, species is stabilized by the presence of less dominant species [2], [24]. Experiments on both natural [25] and theoretical systems [24] suggest that distributions of interaction strengths in species-rich communities are typically skewed towards many weak and few strong species–species interactions.

We could detect only a subtle stabilizing effect of plant species richness on the variance CV of the trace-gas-flux function at the ecosystem level, while the observed stabilizing effect was consistently stronger on ecological functions at the community level (Figure 1). One possibility is that weaker diversity–stability relationships at the ecosystem level are due to the presence of soil compensatory mechanisms, such as inherited carbon pools from previous land management and time-lag responses of the belowground communities. This explanation is supported by at least two recent studies from the Jena Experiment [26], [27], revealing that microbial activity and carbon sequestration in soils were significantly increased only 4 years after the beginning of the experiment. However, we also found that the variance CV of other belowground ecological functions (earthworm biomass production, belowground invertebrate abundances) did not decrease significantly with increasing plant species richness. This suggests a possible link between the nature of the experimental manipulation and temporal stability. In managed grasslands, the belowground rooting system and soil texture remain comparatively unaltered by periodically harvesting the aboveground plant biomass, therefore creating different perturbation regimes.

Following their review of the literature on the diversity–stability hypothesis, Jiang and Pu [7] proposed that temporal stabilization of variables at lower organizational levels could promote stabilization at the higher levels (community, ecosystem); i.e. they proposed what we here called bottom-up effects of species richness on the temporal stability of ecological functions at the community and ecosystem levels of organization. Their proposition is valid even when temporal synchronization remains unaffected with increasing species richness; an assertion largely supported by our results on the co-variance CV (Figures 1 and 2). When different variables characterizing an ecological function are strongly correlated with one another (either positively or negatively), all co-variances reflect the variances. This statistical effect alone could explain the decrease of the co-variance CV of ecological functions with increasing species richness that we observed at the community level (Figure 1), where variables are strongly correlated. Conversely, we observed no effect of increasing plant species richness on the co-variance CV of ecological functions below the community level of organization. This indicates that although a majority of variables characterizing an ecological function at the species and phylogenetic (functional) group levels were in general positively correlated (Figure 2), these variables are not as strongly correlated as those measured at the community and ecosystem levels.

The strong positive correlations among variables measured at the community and ecosystem levels may also result from allometric relationships between plant height, cover and biomass (Plant Stand Structure and Invasive Plant Bioregulation functions), from mathematical relations linking species abundance and richness in ecological communities (Arthropod Diversity function), and from spatial auto-correlation among observations (Soil Water and Nutrient Content functions). Although other factors may cause variables to be more strongly correlated at the higher levels of organization, this should only strengthen the possibility that increasing plant species richness can stabilize ecosystem functions in a bottom-up fashion.

What would cause populations of dominant group of species in a community to co-vary positively (synchronize) in time? Through an extensive review of 41 natural and experimental communities, Houlahan and colleagues [28] concluded that species–environment interactions largely dominate community dynamics, hence driving species populations to co-vary positively in their abundance and biomass. However, it may be premature to conclude whether a positive co-variance indeed reflects the synchronized response of species populations sharing a common set of environmental perturbations [6], [29]. More work will be needed to disentangle whether co-variances are the result of: i) species–species interactions (e.g., synchronous response of several sub-dominant species to a few dominant ones), ii) species–environment interactions (e.g., synchronous response of species to a common set of environmental perturbations), or iii) both interaction types (e.g., synchronous response of consumers to temporal variations in the producers).

Our study advocates a bottom-up effect of plant species populations on the temporal stability of community- and ecosystem-level functions in managed grasslands. Novel conceptual frameworks for the diversity–stability hypothesis should now attempt to go beyond the grouping of ecological functions into species and community levels of organization to fully account for the notion of spatial and temporal observation scale. At small observation scales the population dynamics of a few individuals is captured, while ecosystem dynamics need larger observation scales to consider feedbacks on the population dynamics. Additionally, a more practical quantification of biological interactions is needed that embraces the vast array of species–species competitive and multi-trophic interactions. This will require a better theoretical and empirical foundation of how the pattern of interaction strength between species affects the functioning of whole ecosystems. While managing ecosystem stability in a stochastic and changing world is beyond current abilities [30], preserving or creating diverse ecosystems so far remains our best option to achieve the stabilization of ecosystems.

## Materials and Methods

### Experimental Design

In the Jena Experiment, regularly mown and weeded grassland plots of 20×20 m were established with plant species richness of 1, 2, 4, 8, 16, and 60 species. At each species richness level, 16 experimental plots were created randomly from a pool of 60 species, with the exception of richness levels 16 and 60 for which 14 and 4 plots were available, respectively. Of the measurements conducted in the course of the study, 42 variables were measured on six or more dates and fulfilled the following criteria: the variable contained less than 50% of zeros across all experimental plots and temporal observations, the variable has a temporal variance sufficiently far from zero to prevent the inclusion of processes that do not respond to environmental perturbations or have ceased to operate, and the variable could be temporally aligned along with other variables characterizing the same ecological function. Additionally, for the quantification of belowground invertebrates from Kempson cores (Table 1) two temporal observations were made within a few days in experimental subplots subjected to different treatments: with and without the application of an equal amount of chlorpyrifos.

The 42 variables used in this study were grouped into twelve ecological functions according to their similarity and level of organization (a short description of each ecological function is given in parentheses): 1) Earthworm Biomass production (soil engineering; population level), 2) Parasitic Hymenoptera Abundance (food-web complexity; population level), 3) Invasive Plant Bioregulation (suppression of spontaneously invading plant species; population level), 4) Belowground Invertebrate Abundance (density of dominant soil macrofauna functional groups; phylogenetic clade level), 5) Aboveground Invertebrate Abundance (density of dominant aboveground, mainly saprophagous, phytophagous and parasitic, insect functional groups; phylogenetic clade level), 6) Plant Functional Group Biomass production (aboveground plant productivity; phylogenetic clade level), 7) Plant Stand Structure (vegetation development in cover, height, and biomass; community level), 8) Invasive Plant Bioregulation (suppression of spontaneously invading vegetation in cover, species diversity, and biomass; community level), 9) Arthropod Diversity (arthropod abundance and richness; community level), 10) Soil Water Content (use of water by organisms; ecosystem level), 11) Soil Nutrient Concentration (use of ammonium and nitrate by organisms; ecosystem level), and 12) Trace Gas Fluxes (net ecosystem–atmosphere exchange of CO2, N2O and CH4; ecosystem; ecosystem level).

The main references for the field sampling protocols listed in Table 1 are: Trace Gas Fluxes [31]; Soil Nutrient Concentration [32]; Soil Water Content [33]; Arthropod Diversity, Above-Ground Invertebrates, and Parasitic Hymenoptera [34]; Plant Stand Structure and Plant Functional Group Biomass [16]; Invasive Plant Bioregulation [35]; Below-Ground Invertebrate and Earthworm Biomass [36].

Our primary aim was to include only species, or more generally variables, observed at least once in all experimental plots. This approach has two main advantages. It focuses on a common set of variables that capture the functioning of all grassland plots across the species richness gradient. It avoids confounding the effect of habitat specialists (i.e., sub-dominant species in the present context) with measures of temporal stability at the population level [9]. Furthermore, because plant species were randomly attributed to the different communities in our experiment, each species is only found in a limited number of experimental plots. Therefore, we did not include population variables characterizing the productivity of sown (resident) plant species.

### Temporal stability measures

We linearly scaled variables between zero and one to remove the effect of measurement units. The scaling of a variable *x* was performed across all experimental plots and temporal observations as follows: *x*-min(*x*) /range(*x*). Variables characterizing the Invasive Plant Bioregulation functions (population and community levels), as well as the Soil Water Content and Nutrient Concentration functions (ecosystem level), were expressed in terms of ecological services and were therefore transformed by taking the reciprocal as follows: 1/ (*x*+0.01). Adding 0.01 to these scaled *x* variable prevented division by zero and preserved a log-linear relationship between the squared mean and variance. Then, in each experimental plot, and for each of the twelve ecological functions, we constructed a matrix containing temporal observations in rows and variables in columns. We obtained from each matrix the multivariate coefficient of variation as follows: CV^2^ =  [Σ Variances +Σ Co-variances]/ [Σ Means]^2^. We partitioned the squared coefficient of variation CV^2^ into two additive components: an insurance effect (Variance CV =  [Σ Variances] / [Σ Means]^2^) and a synchronizing effect (Co-variance CV =  [Σ Co-variances] / [Σ Means]^2^). Although summed variances and co-variances have been recently criticized as a means of precisely identifying ecological mechanisms [6], [29], their ability to capture generic aspects of temporal stability in ecosystems is not in question.

The above measures of temporal stability can be defined in the context of previous ecological theories of the diversity–stability hypothesis. An insurance (variance dampening) effect results from functional complementarity among co-occurring species. By increasing the number of plant species, the insurance effect predicts a decrease in the variance CV of ecological functions, which may occur through a differentiation in resource use [1], [4] and a complex network of many weak interactions among species [2], [24]. With increasing species richness, the insurance effect is associated with an increase in the mean level of functionality, which is not exceeded by a similar increase in the sum of variances. On the other hand, the synchronizing (positive co-variance) effect is associated to a temporal coupling among co-occurring species. By increasing the number of plant species, the co-variance CV of ecological functions is predicted to decrease because compensatory interactions intensify among species competing for limited resources [4], [28] or because species are differently sensitive to environmental perturbations [5], [10]. With increasing species richness, the synchronizing effect is associated with a decrease in the co-variances among variables jointly characterizing the same ecological function.
